# Near death experiences: a multidisciplinary hypothesis

**DOI:** 10.3389/fnhum.2013.00533

**Published:** 2013-09-11

**Authors:** István Bókkon, Birendra N. Mallick, Jack A. Tuszynski

**Affiliations:** ^1^Neuroscience Department, Vision Research InstituteLowell, MA, USA; ^2^School of Life Sciences, Jawaharlal Nehru UniversityNew Delhi, India; ^3^Department of Physics, University of AlbertaEdmonton, AB, Canada

**Keywords:** brilliant lights during near death experiences, phosphenes, biophotons, biophysical picture representation, unconscious cognitive processes, rapid eye movement sleep (REMS), out-of-body experience (OBE), quantum entanglements

## Abstract

Recently, we proposed a novel biophysical concept regarding on the appearance of brilliant lights during near death experiences (NDEs) (Bókkon and Salari, 2012). Specifically, perceiving brilliant light in NDEs has been proposed to arise due to the reperfusion that produces unregulated overproduction of free radicals and energetically excited molecules that can generate a transient enhancement of bioluminescent biophotons in different areas of the brain, including retinotopic visual areas. If this excess of bioluminescent photon emission exceeds a threshold in retinotopic visual areas, this can appear as (phosphene) lights because the brain interprets these intrinsic retinotopic bioluminescent photons as if they originated from the external physical world. Here, we review relevant literature that reported experimental studies (Imaizumi et al., 1984; Suzuki et al., 1985) that essentially support our previously published conception, i.e., that seeing lights in NDEs may be due to the transient enhancement of bioluminescent biophotons. Next, we briefly describe our biophysical visual representation model that may explain brilliant lights experienced during NDEs (by phosphenes as biophotons) and REM sleep associated dream-like intrinsic visual imageries through biophotons in NDEs. Finally, we link our biophysical visual representation notion to self-consciousness that may involve extremely low-energy quantum entanglements. This article is intended to introduce novel concepts for discussion and does not pretend to give the ultimate explanation for the currently unanswerable questions about matter, life and soul; their creation and their interrelationship.

## Introduction

An experience of seeing brilliant light is one of the last phenomena in a series of events reported to frequently occur during NDEs. Recently, we suggested that the experience of seeing shining lights in NDEs may be due to the reperfusion that causes overproduction of free radicals that can generate a transient enhancement of bioluminescent biophotons in different areas of the brain, chief among them being visual areas. When bioluminescent photon emission exceeds a threshold, this can lead to the emergence of lights (phosphenes) and the brain interprets these intrinsic bioluminescent photons in visual areas as if they were derived from the external visual world. First, we present important experimental studies reported by several groups (Imaizumi et al., 1984; Suzuki et al., 1985) that support our previously published conception proposing that seeing lights in NDEs may be due to the transient enhancement of bioluminescent biophotons. We briefly present our novel biophysical visual representation concept and theorize that through NDEs, intrinsic visual perceptions and imageries may be due to the rapid eye movement sleep (REMS) associated dream-like biophysical picture representation created from long-term visual memory. Finally, we link our biophysical visual representation notion to self-consciousness that may involve extremely low-energy quantum entanglements by means of biophotons. The aim of this paper is to propose novel ideas rather providing a final explanation of the currently unanswered questions about matter, creation, soul, and life.

## Near death experiences

During NDEs, the subjects' heartbeat and breathing are temporarily suspended, and they exhibit flattened brain waves in the electroencephalogram (EEG) and absence of auditory evoked potentials from the brainstem. NDEs include a wide range of subjective experiences associated with the impending death. NDEs consist of some frequent components such as out-of-body experience (OBE) (separation of consciousness from the physical body), passage through a dark tunnel, encounters with bright lights, meeting deceased relatives, meeting guardian spirits and mystical beings, sensing a border, etc. The subjects' decision of its “SELF” to return into the material body may be voluntary or involuntary (Greyson, 2000; van Lommel et al., 2001; French, 2005; Parnia et al., 2007). NDEs reported by children are similar to those described by adults although their levels of learning and worldly experiences may differ. The characteristics of subjects experiencing NDEs are similar worldwide irrespective of language, culture and age. In general, after NDEs subjects report being happier, less materialistic, more altruistic, and not afraid of death compared with those who did not have this experience, although there have also been reports of fearful experiences similar to nightmares (Agrillo, 2011).

Numerous mechanisms have been hypothesized to elucidate NDEs. The main scientific ideas proposed as interpretations of NDEs phenomena are: temporal lobe malfunctions; imbalances of various neurotransmitters (such as glutamate, noradrenaline, dopamine, endogenous opioids, serotonin); electrolyte disturbances during times of physical crises; REM-sleep intrusions; lack of oxygen in the brain or too much carbon dioxide; similarities between NDEs and effects of hallucinogens; activation of the limbic system; to name a few (Carr, 1981; Persinger and Makarec, 1987; Blackmore and Troscianko, 1988; Appelby, 1989; Jansen, 1989; Lempert, 1994; Blackmore, 1996; Beauregard and Paquette, 2006; Bonilla, 2011; Facco and Agrillo, 2012a).

Let us take a closer look at some examples. Electrical stimulation of the temporo-parietal lobe could induce similar dissociation to that during NDEs and OBEs. Blanke et al. (2002) reported the experience of a 43-year-old woman who had a rare form of epilepsy. These authors used focal electrical stimulation of the brain's right angular gyrus to distort the patient body image and induced OBEs, as well as vestibular and somatosensory responses. During this procedure, the patient thought that she was either larger or smaller or outside her body. Anoxia (Whinnery, 1997) or hypercarbia (abnormally high level of carbon dioxide in the circulating blood) (Meduna, 1950) can produce phenomena such as seeing brilliant lights, reliving past memories and having OBE. The visual cortex dysinhibition that is associated with anoxia (not the anoxia *per se*) has been suggested as an interpretation of tunnel-like perception during NDEs (Blackmore, 1996). Clinical observations support that REMS intrusion can contribute to NDEs (Nelson et al., 2006). Recently, Kevin Nelson (2011) proposed that the common factor in these experiences may be reduced oxygen supply to the brain. If the oxygen supply of the temporo-parietal lobe is cut off, this could start an OBE.

In addition to the neurobiological theories, psychosocial concepts also tried to explain NDEs. These include: expectation hypothesis (when life-threatening situations can initiate NDEs as a projection of expectancy of the afterlife.); depersonalization (during NDEs, depersonalization is a form of detachment including heightened arousal, disorganized emotion, sensation, reality, and experience of time that occurs as a psychological defense against the fear of death); memory of birth (when a baby is born, he/she leaves the womb to travel down a tunnel toward a light and waits for a great deal of love. In contrast, when death is approaching the stored memory contains the events that happened since the individual's life began.); fantasies and imagination (Noyes and Kletti, 1977; French, 2001, 2005; Greyson et al., 2009; van Lommel, 2010). A recent (open access) paper by Facco and Agrillo (2012a) is an excellent summary and evaluation of the various hypotheses proposed to elucidate NDEs.

However, none of the above mentioned concepts itself could elucidate all the reported common characteristics of NDEs. While some features of NDEs may be attributed to neural mechanisms or to psychological phenomena, nevertheless, currently we do not have reasonable explanations of all the reported features of NDEs.

## Phosphenes and brilliant lights during NDEs by bioluminescent biophotons

Biophotons (also referred to as ultraweak (bio)chemiluminescent photons, ultraweak visible spontaneous electromagnetic radiation, etc.) are spontaneous ultraweak photons that are continuously emitted by all living cells and in particular by neurons without external excitation (Tilbury and Cluickenden, 1988; Devaraj et al., 1991; Scott et al., 1991; Cohen and Popp, 1997; Zhang et al., 1997; Takeda et al., 1998; Nakano, 2005; Chang, 2008; Kobayashi et al., 2009; Rahnama et al., 2011). Biophotons originate from natural bioluminescent radical reactions and the deactivation of energetically excited molecules. Neurons also constantly produce biophotons through bioluminescent radical reactions during normal metabolism (Isojima et al., 1995; Kataoka et al., 2001). In addition, the intensity of biophoton production in the rat brain *in vivo* has been correlated with cerebral energy metabolism, EEG activity, cerebral blood flow, and oxidative processes (Kobayashi et al., 1999a,b), which suggests that there is neural activity-dependent biophoton emission taking place in the brain (Isojima et al., 1995).

Phosphenes represent a perceived sensation of flashes of light in the absence of external visual stimulation. The most common phosphenes are pressure phosphenes, caused by rubbing the closed eyes. Earlier, we have proposed (Bókkon, 2008) that the phosphene phenomenon is due to the intrinsic perception of induced (mechanical, electrical, magnetic, etc.) or spontaneous increased bioluminescent biophoton emission of cells in various parts of the retinotopic visual system. Induced or spontaneous unregulated overproduction of free radicals and energetically excited molecules can create a brief increase of the generation of bioluminescent biophotons in the visual system. When this excess biophoton emission can exceed a threshold, they appear as phosphene lights in the subject's mind.

Our hypothesis that phosphene lights are due to biophotons is supported by several sets of experiments. Catalá (2006) has shown that radicals from lipid peroxidation of the photoreceptors can create (bio)chemiluminescent photons (bioluminescence is a type of chemiluminescence, which naturally occurs in living organisms) in the visual spectrum. Subsequently, our prediction regarding one specific kind of phosphenes (i.e., retinal phosphenes during space travel) was supported by Narici et al. (2009). According to this latter work, ionizing radiation (cosmic particle rays) induced free radicals which produce chemiluminescent photons through processes including by lipid peroxidation. Chemiluminescent photons are then absorbed by the photoreceptors and initiate a photo-transduction cascade, which results in the perception of phosphenes. Narici et al. (2012) also revealed that the lipid peroxidation of the photoreceptors can produce (bio)chemiluminescent photons that generate anomalous visual effects, such as those associated with retinal phosphenes. Recently, the first experimental *in vitro* evidence was presented (Wang et al., 2011) for the existence of spontaneous and visible light induced biophoton emission from freshly isolated whole eye, lens, vitreous humor, and retina samples from rats. It also supports the hypothesis that phosphene lights are produced by biophotons. Since phosphenes can be produced by direct stimulation of the visual cortex without a retinal photo-transduction cascade, this suggests that retinal and visual cortical phosphenes are generated by similar mechanisms, and both may be due to the transiently and locally increased ultraweak biophotons.

Several experiments demonstrated that during post-ischemic reperfusion, there is considerable overproduction of oxygen free radicals generated in the brain and the retina (Agard et al., 1991; Ophir et al., 1993; Basu et al., 2011). In the recovery phase, the overproduction of free radicals and excited species in the visual areas, among others, can produce significant bioluminescent biophotons by means of lipid peroxidation.

Based on the above-mentioned experiments and notions generated by their interpretation, we proposed an original biophysical hypothesis regarding the appearance of brilliant lights during NDEs (Bókkon and Salari, 2012). In particular, we suggested that perceiving shining lights during NDEs may be due to bioluminescent biophotons simultaneously produced in the recovery period in several areas of the visual system and the brain interprets these biophotons as if they originated from the external visual world. It means that brilliant light experiences in NDEs can be simply interpreted as simultaneously produced phosphenes (biophotons) in numerous visual areas during the recovery phase.

To test the validity of our idea we suggested (Bókkon and Salari, 2012) that *in vitro* or *in vivo* increases of ultraweak bioluminescent photon emission should be measured from hemispheres in *in vivo* animal experiments before, during and after the recovery period during experimental cardiac arrest. However, through an extensive literature search, we have found relevant experimental studies (Imaizumi et al., 1984; Suzuki et al., 1985) that support our previously formulated hypothesis regarding the appearance of brilliant lights during NDEs.

## Ultraweak (bio)chemiluminescence in hypoxic brain: correlation between energy metabolism and free radical reaction

In 1984 Imaizumi et al. determined the ultraweak (bio)chemiluminescence value at pre-hypoxia, during hypoxia and at post-hypoxia states in rat brains. Brain hypoxia was produced by arterial hypoxemia (PaO2 17–22 mmHg), normocapnia (PaCO2 28–38 mmHg) and normotension (MABP 100–140 mmHg). Rat brain samples were collected at pre-hypoxia, at 3 and 5 min during hypoxia and at 5 and 30 min during post-hypoxia states.

Ultraweak chemiluminescence values were 11 ± 15 counts/10 s-g in pre-hypoxia state, risen to 231 ± 35 counts/10 s-g at 3 min, slightly decreased to 154 ± 62 counts/10 s-g at 5 min of hypoxia, and rose to 217 ± 79 counts/10 s-g at 5 min of posthypoxia. Finally, at 30 min of post-hypoxia, values returned to low levels 10 ± 13 counts/10 s-g which are similar to the prehypoxic values. Chemiluminescence spectral peaks of intensity were found at 480, 520–530, 570, 620–640 and 680–700 nm. This suggests that ultraweak luminescence originated from singlet oxygen species. Regarding the energy metabolism, during the hypoxic state, ATP (Adenosine 5′-triphosphate) and glucose exhibited a slight decrease, while ADP (Adenosine 5′-diphosphate) showed an increase that suggests disorders in the tricarboxylic acid cycle (TCA), glycolysis, and mitochondrial oxidative phosphorylation. In the post-hypoxic state all metabolites were recovered at 30 min, which suggests that brain hypoxia was reversible. Suzuki et al. (1985) suggested that the major source of free radicals originated from lipid peroxidation, since the ischemic brain is associated with membranous lipid peroxidation.

In the second series of experiments (Suzuki et al., 1985), (under circumstances similar to those in the first experiments, Imaizumi et al., 1984) researchers evaluated the effect of pre-treatment with protective drugs such as vitamin E, betamethasone and mannitol on free radical reactions in hypoxic rat brain tissue by ultraweak chemiluminescence measurements. Pre-treatment with vitamin E and betamethasone diminished all chemiluminescence intensity peaks, but little decrease occurred after mannitol was administered. These results indicate an *in vivo* free radical scavenging effect of these drugs.

### Implications of the results of imaizumi et al. and suzuki et al.

- Experiments by Imaizumi et al. (1984) and Suzuki et al. (1985) support our notion (Bókkon and Salari, 2012) that perceiving lights during NDEs may be due to bioluminescent biophotons that originate from unregulated overproduction of free radical bioluminescent biochemical reactions and the brain interprets these biophotons as if they originated from virtually the external visual world.
- Perturbation of mitochondrial oxidative phosphorylation also supports our hypothesis, because mitochondria are major sources of free radicals and biophotons (Thar and Kühl, 2004).
- Ultraweak (bio)chemiluminescence spectral intensity peaks were observed at 480, 520–530, 570, 620–640 and 680–700 nm. However, there were significant individual differences in measured intensity of ultraweak (bio)chemiluminescence during and after hypoxia states. This can explain the fact that some subjects experienced light perception but other subjects did not experience this during NDEs. In particular, the individual brain structures and the individual oxidative metabolic processes may explain which person can remember the lights and which cannot remember them during NDEs.
- We also proposed that lights in NDEs may be experienced in the recovery period in several areas of the visual system. In contrast, experiments by Imaizumi et al. (1984) and Suzuki et al. (1985) revealed that increased ultraweak (bio)chemiluminescence can emerge after the induction of hypoxia states and this increased biophoton production can persist throughout all hypoxia and post-hypoxia (reperfusion) states, i.e., up to 30 min after reperfusion when values returned to low levels 10 ± 13 counts/10 s-g that were similar to the prehypoxic values. Although it is likely that seeing lights in NDEs may occur in the reperfusion state, experimental outcomes by Imaizumi et al. (1984) and Suzuki et al. (1985) also suggest that the light sensing may occur at any state in NDEs.

## Intrinsic visual sensation and imagery by ultraweak (bio)chemiluminescence in NDEs

Since enhanced ultraweak (bio)chemiluminescence can appear after the induction of hypoxia states and this increased biophoton emission can persist throughout all hypoxia and post-hypoxia (reperfusion) states, this raises a further possibility regarding NDEs. Namely, NDEs consist of some recurrent components among which meeting deceased relatives, meeting guardian spirits and mystical beings or sensing a border are common occurrences. These intrinsic visual experiences during NDEs can be linked to our recently suggested biophysical visual representation concept (Bókkon, 2009; Bókkon and D'Angiulli, 2009).

According to our novel biophysical representation idea (Bókkon, 2009; Bókkon and D'Angiulli, 2009) objects in the visual field can be directly represented in the retinotopically organized neural networks of striate cortex (also known as primary visual cortex or V1) by congruent patterns of biophotons generated from regulated bioluminescent radical/redox processes, and iterative computation (Bókkon et al., 2011a). Our concept of intrinsic biophysical visual virtual reality by bioluminescent photons in early retinotopic areas may simply be a first possible biophysical basis of Kosslyn's depictive pictorial theory (Kosslyn, 1994; Lewis et al., 2011; Cichy et al., 2012). It claims that visual perception and imagery share common neural substrates, and that both visual perception and imagery induce activation in retinotopically organized striate and extrastriate regions.

Specifically, photons reflected from objects are absorbed by photoreceptors and converted into retinal electrical signals. Next, retinotopic electrical signals are conveyed to the V1, where spike-related electrical visual signals are induced along classical axonal-dendritic pathways. These spike-related electrical visual signals travel along classical axonal-dendritic pathways and concurrently produce spike-related (neural activity-dependent) biophotons within the same population of retinotopic V1 *neurons* through mitochondrial redox processes. These synchronized and activity-dependent biophotons can spatially and temporally create intrinsic pictures in the early visual area. Thus, retinal visual information can be re-represented through regulated biophotons in retinotopically organized, mitochondrial cytochrome oxidase-rich visual areas during visual imagery, visual perception as well as during REMS associated dreams or visual hallucination.

**Figure 1:**
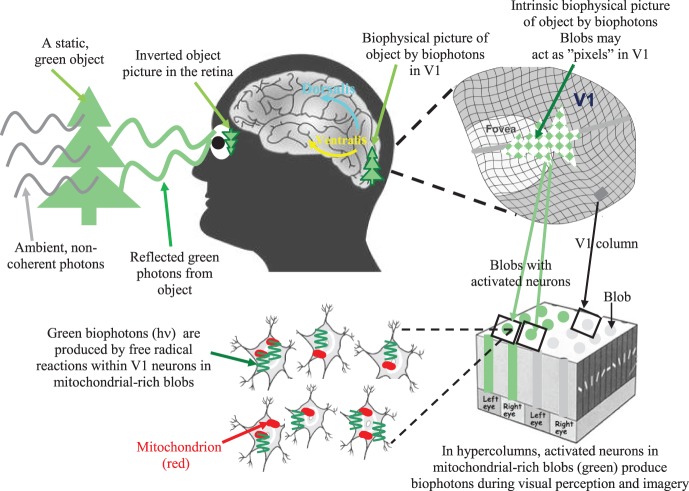
**A simple illustration of the biophysical picture representation idea (also called intrinsic biophysical virtual visual reality) during visual perception and imagery (Bókkon, 2009; Bókkon and D'Angiulli, 2009).** External photon signals reflected from an object are converted into retinotopic electrical signals inside the retina. Next, retinotopic electrical signals are conveyed to the V1 and transformed into controlled biophotons by mitochondrial redox processes within the V1 neurons. Specifically, spike-related, retinotopic electrical signals create synchronized biophotons along classical axonal-dendritic pathways through a redox reaction within retinotopic V1 neurons. Small groups of visual neurons can function as “visual pixels” that are appropriate to the topological distribution of the retina's photonic signals. Thus, we can get an intrinsic computational biophysical picture of the object created by biophotons in the retinotopic V1. The long-term visual information is not stored as pictures but as epigenetic codes. We are able to identify objects since the same epigenetic processes are activated every time we see an object. Therefore, the representation stored in long-term visual memory will match the representation that is created while the object is seen again. Top-down mechanisms control the epigenetically encoded, long-term visual information during visual processing. Then, according to this retrieved epigenetic information, synchronized retinotopic neurons generate dynamic patterns of biophotons through redox reactions. Finally, biophotons within the millions of synchronized neurons (Bókkon et al., 2011a,b) can create biophysical pictures in the early retinotopic visual area. During visual perception and imagery, visual information is linked and combined with different sensory modalities and higher-order associational areas during multisensory interactions. It should be emphasized that neural electrical signals are transmitted between neurons, but biophotons are generated within retinotopic visual neurons. In addition, intrinsic biophysical pictures (images) are not like rigid objects but we can alter images *ad-lib*, which make it possible that the visual system can also produce irrationally assembled pictures and scenes during REM dreams and visual hallucinations.

Small groups of retinotopic V1 neurons with biophotons might act as “nonlinear visual pixels” with respect to the topological distribution of photonic signals in the retina. As a result, we can obtain a biophysical picture of the objects created through biophotons in the retinotopic V1 (see Figure 1). Our theory implies that there could be a literal image, albeit abstract, in the visual brain's neurons of which the subjects are conscious, and biophotons act as the physical substrate of its subjective experience.

The visual content of biophysical representations generated from regulated biophotons is progressively degraded during the transmission along pathways from V1, V2 (extrastriate visual cortical area), and additional visual areas to higher-level associative regions. Furthermore, higher-level cognitive processes might become progressively more abstract or schematic. The biophysical hypothesis suggests that binding between analogic-perceptual and propositional-abstract formats might appear as a natural consequence of the dynamic “crosstalk” between the visual system and the rest of the brain.

Long-term visual memories are not stored as biophysical pictures but as redox regulated epigenetic codes^1^. During visual imagery, top-down processes trigger and regulate the epigenetic encoded long-term visual information. Next, according to retrieved neural epigenetic information, mitochondrial networks in synchronized neurons generate patterns of biophotons through redox reactions, which can produce intrinsic biophysical pictures in retinotopic and mitochondrial rich visual neurons during visual imagery, REMS associated dreams or visual hallucination (Bókkon, 2009; Bókkon and D'Angiulli, 2009; Bókkon et al., 2010a). First biophoton experiments may support our biophysical pictures representation. Namely, Dotta and Persinger (2011) and Dotta et al. (2012) observed cognitive coupling with biophoton emission in the brain during subjective visual imagery. In addition, the biophoton emissions were strongly correlated with EEG activity and the emergence of action potentials in axons. In addition, Sun et al. (2010) revealed that biophotons can conduct along the neural fibers which supports our biophysical picture hypothesis. It appears that biophotonic and bioelectronic activities are not independent biological processes in the nervous system, and their synergistic action may play a significant role in neural signal processes.

One might argue that neurons in early retinotopic visual areas could be activated by biophotons released as a consequence of spiking activity. We should stress that neural electrical signals (spiking activity) run along retinotopic visual neurons, but external neural biophotons cannot activate other neurons because visible biophotons can be easily absorbed within neural cells. Thus, the emergence of intrinsic biophysical pictures is due to the synchronized biophoton production created within retinotopic neurons during synchronized neural electrical discharges. In addition, our calculations (Bókkon et al., 2010b) indicate that the biophoton intensity within retinotopic neurons might be sufficient for creating a biophysical pictorial representation of a single-object during visual perception.

One might also argue that there does not exist an analog for the photon-dependent 11 cis-trans retinal conversion that occurs in retinal photoreceptors occurring in populations of cortical neurons, which could then account for perceptual representation of such events. However, the emergence of intrinsic biophysical pictures is not due to the conversion of biophotons but to the synchronized production of biophotons within retinotopic visual V1 neurons. In other words, there is an intrinsic biophysical picture re-representation of perceived visual scene (reflected photons from an external object) by means of synchronized production of biophotons in retinotopic V1.

Regarding our biophysical visual representation idea, it should be stressed that the phrase “ultraweak biophoton emission” is confusing, as it suggests that ultraweak biophotons are not important in cellular mechanisms but are by-products of free radical reactions. In contrasts, it is plausible that externally measured ultraweak biophoton emission from cells and neurons is principally produced from natural oxidation processes on the surfaces of cellular membranes as demonstrated by Blake et al. (2011). However, the real biophoton intensity can be fundamentally higher inside cells and neurons (Bókkon et al., 2010b) compared with the biophoton intensity in their surrounding environment which makes it possible for the emergence of intrinsic biophysical pictures in V1 visual areas.

### REMS associated dream-like biophysical intrinsic visual imageries during NDEs

Some clinical studies suggest that REMS intrusion may contribute to NDEs and that near-death experiences are likely to mix up (lucid or conscious) dreams and reality (Nelson et al., 2006). Recently, we argued (Bókkon and Mallick, 2012) that activation of the retinotopic visual areas is central to REMS associated dreams and that REMS associated dreaming and visual imagery may have co-evolved in homeothermic animals during evolution. In addition, visual imagery during REMS utilizes a common visual neural pathway similar to that used in wakefulness and during dreams expressed during REMS (Braun et al., 1998; Cantero et al., 1999; Gottesmann and Gottesman, 2007; Miyauchi et al., 2009; Horikawa et al., 2013). This pathway subserves visual processes accompanied by auditory experiences and intrinsic feelings. We also suggested earlier that a protoconscious state manifested during REMS, which may be compared with that introduced by Hobson (2009) many years ago, may be a basic visual process. According to Thonnard et al. (2013), “NDE memories have more characteristics than any kind of memory of real or imagined events and of other memories of a period of coma or impaired consciousness following an acquired severe brain dysfunction.” These authors suggested that the physiological origins of NDEs possible are hallucinations or dream-like events that have rich characteristics like memories of real events.

Concerning our biophysical picture representation idea, during NDEs, intrinsic visual perceptions and imageries may be due to the REMS associated dream-like biophysical biophotonic representations originated from long-term visual memory. During NDEs, these REMS associated dream-like biophysical biophotonic representations may occur in the reperfusion state but are also possible at any state of NDEs.

One might argue that the experience of light can also occur under circumstances that have nothing to do with heart-stopping, anoxia, etc. (Cardeña, 2005; Facco and Agrillo, 2012b). The term entoptic phenomena (subjective visual phenomena) refer to visual (light) experiences derived from within the eye or brain that are not due to the external perception of visible photons in normal vision (Tyler, 1978; Lewis-Williams and Dowson, 1988). Phosphenes, form constants (form constants are complex and reproducible phosphenes) and more complex visual hallucinations are entoptic phenomena that are not only associated with emotional factors, drugs, alcohol, stress, fever or psychotic conditions (Cervetto et al., 2007) and can be early symptom of a variety of diseases of the retina and the visual pathways, but healthy individuals can perceive them as well (Lewis-Williams and Dowson, 1988; Bókkon, 2008). Phosphene lights can be elicited by various stimuli (mechanical, electrical, magnetic, etc.) of cells in the visual systems as well as random firing of cells in the visual areas (Reznikov, 1981; Lindenblatt and Silny, 2002; Merabet et al., 2003). The briefly described concept of phosphenes (Bókkon, 2008) together with the intrinsic biophysical visual pictures by bioluminescent photons during visual perception and imagery, as well as in visual REMS associated dreams and visual hallucination, is able to explain the subjective sensation of light that can also occur under circumstances that have nothing to do with heart-stopping, anoxia, etc. (Bókkon, 2009; Bókkon and D'Angiulli, 2009; Bókkon et al., 2010b). Namely, our intrinsic biophysical visual picture representation concept is not restricted to visual phenomena during NDEs, but can present a common and convergent interpretation for entoptic visual phenomena during normal, pathological, NDE-like and in NDEs circumstances.

In addition, it is possible that entoptic visual phenomena such as phosphenes, form constants and complex visual hallucinations are due to transient or continuous deafferentation and disintegration of certain visual structures that produce an increase in excitability of deafferented neurons (Burke, 2002). This deafferentation can be linked to an increase in spontaneous activity and synchronization of nerve discharges. Thus, visual hallucinations may be considered as local paroxysms in visual structures that can produce dream-like pictures by bioluminescent biophotons in the retinotopic visual areas. These unregulated dream-like pictures by bioluminescent photons can then break into the waking consciousness (Bókkon, 2005; Gottesmann and Gottesman, 2007).

### Evanescent brain processes in NDEs?

Chawla et al. (2009) reported observations involving patients who were neurologically intact before the decision to withdraw life-support devices due to general systemic critical illness. EEG monitoring devices were placed on the patients' forehead. When life-support devices were switched off and blood pressure of these patients was stopped, the monitored EEG activity began to decline. However, declining EEG activity was followed by a short-lived (from 1 to 5 min) but high intensity transient spike in EEG activity. In a transient EEG surge, a high frequency (high frequency gamma oscillations) waveform emerged that is generally related to consciousness. Chawla et al. (2009) proposed that the resuscitated patients could recall their experiences related to the EEG surge that appear similar to what a large number of people described in NDEs. This study can be criticized for its methodology since the subjects were dying and were not resuscitated. Consequently, there is no information available whether the subjects had experienced anything at all.

According to Hameroff and Chopra (2010a), the observed gamma oscillations reported in the studies of Chawla et al. (2009) can be linked to consciousness states that involve particularly low-energy quantum entanglements that persisted over time while other brain functions have run out of energy supply. Consciousness could persist outside the physical body but remains localized on the level of Planck-scale geometry (Hameroff and Chopra, 2010b). “A quantum basis for consciousness also raises the scientific possibility of an afterlife, of an actual soul leaving the body and persisting as entangled fluctuations in quantum spacetime geometry” (Hameroff and Chopra, 2010b). When the patient's physical body is resuscitated, the quantum information can reenter it, and the subjects may be able to recall their experience involving NDEs.

Previously, we emphasized (Bókkon and Vimal, 2010) that retained, subliminal visual representation processes cannot be detected by even the most modern neural recording procedures, but require active stimulation to emerge. This active stimulation can be performed by artificial (external) stimulations, such as TMS (transcranial magnetic stimulation), or by natural (internal) stimulations, like active visualization processes. The idea that evanescent processes cannot be revealed by means of the most modern neural recording procedures may be also applicable to the case of EGG in NDEs. Namely, in regard to the flat EEG in NDEs, EEG determines electric neural activity that occurs very poorly below the upper layers of the cortex. Signal-to-noise ratio is also very low. Spontaneous activity is usually considered to be noise if one is interested in stimulus processing and the level of error increases with the depth below the surface of the cortex (Šobajic, 2002). Since EEG only measures the surface of the brain function, there could be deeper processes taking place that we are unaware of at the present time. Hence, the flat EEG in NDEs does not mean that evanescent brain processes cannot be realized, we simply cannot rule out this possibility (Agrillo, 2011).

Very recently, Borjigin et al. (2013) recorded EEG signals over the frontal, parietal, and occipital cortices bilaterally in rats during wakefulness, anesthesia, and cardiac arrest. Within the 30 s after the rats' hearts stopped beating it was revealed that cardiac arrest produced a transient and global surge of synchronized gamma oscillations of brain activity that exceeded the waking state. In addition, researchers found the high levels of global alpha–gamma coupling that suggest the visual cortex can be highly activated in cardiac arrest. Previous studies indicated that alpha–gamma coupling is especially important for visual perception (Spaak et al., 2012). Dr Jimo Borjigin said (Morelle, 2013), “… it was feasible that the same thing would happen in the human brain, and that an elevated level of brain activity and consciousness could give rise to near-death visions.” “The fact they see light perhaps indicates the visual cortex in the brain is highly activated—and we have evidence to suggest this might be the case, because we have seen increased gamma in area of the brain that is right on top of the visual cortex.” The results of these experiments are consistent with our biophysical picture representation idea during NDEs (Bókkon, 2009; Bókkon and D'Angiulli, 2009; Bókkon et al., 2010a).

To summarize it briefly, it is probable that during NDEs, the increased biophoton production (Imaizumi et al., 1984; Suzuki et al., 1985) can occur at any state in NDEs. In addition, declining EEG activity was followed by a short-lived (from 1 to 5 min) but high-intensity transient spikes in EEG activity (Chawla et al., 2009) when life-support devices were switched off and blood pressure of patients was stopped. In addition, according to the experiments of Kobayashi et al. (Kobayashi et al., 1999a,b), *in vivo* imaging of spontaneous biophoton emission from a rat's brain correlated with cerebral energy metabolism, EEG activity, cerebral blood flow, and oxidative stress. Moreover, Isojima et al. (1995) reported neural activity-dependent biophoton emission from hippocampal slices of rat brain. Thus, the biophoton emission of neurons is in direct correlation with biochemical processes of neurons, i.e., there is a neural activity-dependent biophoton emission in the brain. The high-intensity transiently synchronized gamma oscillations in EEG activity (Chawla et al., 2009; Borjigin et al., 2013) may also reflect an increased biophoton production in NDEs.

Our biophysical visual representation idea may explain not only brilliant lights experienced during NDEs (by phosphenes as biophotons) and REMS associated dream-like intrinsic visual perceptions and imageries through biophotons in NDEs, but also can be linked to the idea by Hameroff and Chopra (2010b) since consciousness involves extremely low-energy quantum entanglements that can return to the material body if the person is resuscitated.

## Out-of-body experience and out-of-body-like experience

OBE is a common experience that occurs, most notably with NDE. During an OBE, people are in an awake-like state and feel that their self or awareness is placed outside of their physical body and rather elevated. The various proposed scientific explanations (see a good summary paper by Neppe, 2011) try to relate the OBE phenomenon to different dysfunctions and pathologies of the brain that are caused by stroke, autoscopy, epilepsy, drug abuse, traumatic experiences such as car accidents, etc., or by artificial electrical stimulation of the brain's angular gyrus stimulating illusory own-body perceptions (Blanke et al., 2002). For instance, Ehrsson (2007) induced an illusion of being outside the physical body in healthy voluntaries by means of manipulation of visual and tactile perceptions. However, in many cases during OBE experience, people involved in them could recall and report specific details of events that have taken place when they had been unconscious (van Lommel et al., 2001; van Lommel, 2006). Although neurophysiological processes must take part in various organs of a person involved in an NDE, in the cases when people could account about specific details of events that have taken place when they had been unconscious, this challenges currently accepted conventional medical science.

The OBE component of NDEs actually offers an opportunity to determine the relationship between consciousness and brain function as well as if there is self-consciousness outside of the physical body during NDEs, although in this latter case it is very difficult to obtain concrete and reliable results. In 2008 the AWARE (Agrillo, 2011; see also the AWARE link in References) study was launched by the Human Consciousness Project in which 25 hospitals took part in Europe and North America via international collaboration of scientists, physicians, and nurses studying subjects who could survive cardiac arrest and report about a NDE. A clever idea was implemented in AWARE as special shelving was placed in resuscitation areas and images were put on shelves that could only be seen from above. If a patient could see (report) the picture, it would indicate whether or not the patient's experiences were illusions or false memory, or if there was indeed self-consciousness present outside of his/her body during OBE. At the moment, AWARE scientists unfortunately are not able to release any information until the conclusion of the study but indications have been given that the results, obtained during the first five years, can be released throughout 2013 through appropriate scientific publications.

Nevertheless, we should make a phenomenological distinction between OBE and out-of-body-like experience (Neppe, 2011). The latter can be explained by diverse forms of dysfunction and pathologies of the brain or can be produced in healthy persons in the laboratories, but the former cannot be explained yet but may be related, for example, to quantum mechanisms. So, we may definite the OBE if a person could report about specific details of events that have taken place when they had been unconscious, and person's narrative can be genuinely checked afterwards similar to goal of the mentioned Human Consciousness Project.

## Some reflections: extending the idea of hameroff and chopra related to NDEs

Recently, we have suggested that characteristics of homeothermic states make the development of explicit memory possible in evolution (Bókkon, 2005). Our idea appears to be related to Hobson's protoconscious notion (2009), i.e., protoconscious state may emerge from implicit memory in homeotherms during the evolution of REMS. We also suggested that the REMS protoconscious state may be basically a visual process and REMS associated visual dreams and visual imagery may have co-evolved in homeothermic animals in evolution (Bókkon and Mallick, 2012).

It is possible that the appearance of self-consciousness in humans is due to the emergence of a very well structured neocortex and the development of language. According to Hassin (2013), “unconscious processes can perform the same fundamental, high-level functions that conscious processes can perform.” van Gaal et al. (2008) stated that “unconscious stimuli can influence whether a task will be performed or interrupted, and thus exert a form of cognitive control.” In addition, although the neural correlates of consciousness have traditionally assigned a key role to the prefrontal cortex generating consciousness and high-level conscious control, present neuroscientific experiments reveal that prefrontal cortex can be activated unconsciously (van Gaal et al., 2008) and challenge the fundamental function of the prefrontal cortex in consciousness (van Gaal and Lamme, 2012). It seems that definite brain regions (cognitive modules) can support specific cognitive roles but that consciousness is independent of this (van Gaal and Lamme, 2012). These findings challenge traditional views concerning the proposed relationship between awareness and cognitive control and stretch the alleged limits and depth of unconscious information processing.” Moreover, according to recent studies, when the subject's decision reached awareness it had been influenced by unconscious brain processes for up to 10 s (Soon et al., 2008; Bode et al., 2011; Soon et al., 2013). However, future studies should reveal if unconscious and conscious decisions and representations may share common neural processes and substrates.

We proposed that the human unconscious can operate through intrinsic dynamic biophysical pictures and we link these picture-representations to each other during language learning processes (Bókkon, 2009; Bókkon and D'Angiulli, 2009; Bókkon et al., 2011a). Thus, the human self-consciousness in the waking state may be an abstract language dependent manifestation of the unconscious. Our thinking processes and indeed every decision made at a given moment can be a coherent and convergent dynamic (discrete events) manifestation of our unconscious cognitive (essential picture-representation) processes due to the billions of non-conscious processes.

Popper et al. (1993) stated: “I wish to propose here as a hypothesis that the complicated electro-magnetic wave fields which, as we know, are part of the physiology of our brains, represent the unconscious parts of our minds, and that the conscious mind—our conscious mental intensities, our conscious experiences—are capable of interacting with these unconscious physical force fields, especially when problems need to be solved that need what we call “attention.” This admittedly vague working hypothesis appears to me as a small yet significant progress within a so far hopelessly difficult part of physiology.” It seems that Popper's notion (electro-magnetic wave fields represent the unconscious parts of our minds and that the conscious minds are capable of interacting with these unconscious physical force fields) is very similar to our biophysical representation concept by ultraweak electromagnetic biophotons.

The idea by Hameroff and Chopra (2010b) that the self-consciousness could continue outside the body but remains at a level of Planck-scale geometry (Planck length is about 10^−33^ cm), may be realized by potential quantum-like properties of biophotons (and by virtual photon particles, see in Bókkon, 2003). We hypothesize that human self-consciousness is an individual-specific abstract manifestation of language and experience-dependent expression of conscious plus unconscious exposure and experiences of an individual. Here we talk about self-unconsciousness that performs cognitive processes through intrinsic dynamic biophysical pictures. Extending the idea of Hameroff and Chopra (2010b) (“A quantum basis for consciousness also raises the scientific possibility of an afterlife, of an actual soul leaving the body and persisting as entangled fluctuations in quantum space-time geometry.”), we propose that self-consciousness originates within the core of the living body having all the metabolic properties intact. The latter having wave properties possesses the potential to propagate and thus, may manifest around the physical limits of the body.

Furthermore, we hypothesize that in the cases of NDE and OBE, because of the decreasing cellular metabolic activities, the internal as well as external inputs to the brain (cortex) are significantly reduced and therefore, the self-consciousness perceives and manifests other experiences. This gives the impression that the cognitive processes of the individual continue outside the body and if the body can be resuscitated, the quantum-like self-unconsciousness re-enters the physical body and the subjects may be able to recall and report their experience in terms of NDEs, which are modulated by their idiosyncrasies. If resuscitation happens to be unsuccessful and the subject dies, then self-unconscious [or implicit self (soul) awareness] as an entity may return into the subspace void where it blends and combines with the self-sustaining creation. This, however, cannot be reported back to the mortal living beings for obvious reasons.

The most ancient philosophical and conceptual works, the *Upanishads* claim that the mind experiences self through extra-fine thread like connections, the “*nadi*,” which has been defined as finer than a thousandth part of a hair and it carries different “*hues*” of varying intensity (Mallick and Mukhopadhyay, 2011). The dimension of the latter is in the range of nanometers, which is practically the unit of wavelength of visible light photons (300–700 nm). It is interesting that this ancient concept may be explained by assuming that the mind and self-communicate through bioluminescent biophotons, which may support our proposed biophysical concept (Bókkon, 2009; Bókkon et al., 2010a, 2011a,b) that intrinsic biophysical pictures (also referred to as biophysical visual virtual reality) can emerge during visual imagery associated to dreams during REMS.

The above mentioned notions are consistent with Hameroff's ideas (2012) expressed in the following statements: “Support for consciousness as sequences of discrete events is also found in Buddhism, trained meditators describing distinct “flickerings” in their experience of pure undifferentiated awareness (Tart, 1995, pers. communication). Buddhist texts portray consciousness as “momentary collections of mental phenomena,” and as “distinct, unconnected and impermanent moments which perish as soon as they arise.” Buddhist writings even quantify the frequency of conscious moments. For example, the *Sarvaastivaadins* (von Rospatt, 1995) described 6,480,000 “moments” in 24 h (an average of one “moment” per 13.3 ms, 75 Hz), and some Chinese Buddhism as one “thought” per 20 ms (50 Hz), both in gamma synchrony range” and description of comparable concepts may be found in still earlier *Vedic, Upanishadic* and other *Hindu* philosophic scriptures.

## Short summary

Here we presented some novel ideas to stimulate new concepts that may facilitate the understanding of the phenomena of NDEs. First, we reviewed and evaluated relevant literature that reported experiments (Imaizumi et al., 1984, Stroke; Suzuki et al., 1985, Stroke) that support our previously published conception, i.e., that seeing lights in NDEs may be due to the transient enhancement of bioluminescent biophotons. Then, we described our biophysical visual representation notion and theorized that through NDEs, visual imageries may be due to the REMS associated dream-like biophysical picture representation created from long-term visual memory. This is certainly a complex process, however, understanding the neurophysiological and neurochemical substrates of REMS regulation is likely to provide insights and eventually a better understanding of this phenomenon. However, Agrillo (2011) raised an crucial question regarding NDEs, “It is worth noting that most of the recurring features are visual experiences (seeing a light, seeing a tunnel, deceased people, or heavenly or hellish landscapes). This raises an interesting question: why would an out-of-body mind still perceive the reality mainly driven by visual processes?” Our briefly described biophysical visual representation concept may present a possible answer to the question why most of the recurring features are visual experiences during NDEs.

Hameroff and Chopra (2010b) speculated that self-consciousness involves extremely low-energy quantum entanglements that could return to the material body if the person is resuscitated. We linked our biophysical visual representation notion to self-consciousness that may involve extremely low-energy quantum entanglements by means of biophotons.

It is interesting to note that the *Upanishads* that are the most ancient philosophical and conceptual works containing the mature wisdom of the East can be seen to be consistent with our recently proposed biophysical concept that intrinsic biophysical pictures may appear by regulated biophotons during visual imagery and REMS associated dream visual imagery.

Although many phenomena of NDEs may be explained scientifically, however, phenomenon such as the OBE is not likely to be explained by mere conventional physical and neurological processes. Nevertheless, the final explanations involving the conscious mind, subconscious, matter, life, soul, and the creation are currently unavailable. According to Facco and Agrillo (2012a), “It is now time to remove the ongoing cultural filters and include consciousness, spirituality, and the highest mind expressions in neuroscience in a free, secular, and scientific perspective to overcome old prejudices.”

### Conflict of interest statement

The authors declare that the research was conducted in the absence of any commercial or financial relationships that could be construed as a potential conflict of interest.

## Footnotes

^1^ There is increasing experimental evidence that epigenetic modifications *(i.e., the regulation of chromatin structure through methylation of DNA or post-translational modification of histone proteins, including methylation, acetylation, and phosphorylation)* within neurons are fundamental mechanisms for the formation and storage of behavioral memory (Nelson and Monteggia, 2011; Lipsky, 2013; Zovkic et al., 2013; Rudenko and Tsai, 2013). Most recent studies imply that epigenetic modulation of the genome is a necessary for the neuronal plasticity and long-term memory (Feng et al., 2007). Chromatin arrangement can represent a memory and allow for temporal integration of spaced signals or metaplasticity of synapses (Levenson and Sweatt, 2005). According to the epigenetic concept, cognitive and memory functions are performed not only by neural networks but also by intrinsic processes of neurons (Arshavsky, 2006). The synaptic plasticity (neural networks) concept does not contradict the epigenetic idea, because networks can operate as constantly variable information channels among neurons, but long-term memory can have epigenetic nature in individual neurons.
